# Mathematical modelling and deep learning algorithms to automate assessment of single and digitally multiplexed immunohistochemical stains in tumoural stroma

**DOI:** 10.1016/j.jpi.2023.100351

**Published:** 2023-11-19

**Authors:** Liam Burrows, Declan Sculthorpe, Hongrun Zhang, Obaid Rehman, Abhik Mukherjee, Ke Chen

**Affiliations:** a Department of Mathematical Sciences and Centre for Mathematical Imaging Techniques, University of Liverpool, Liverpool, United Kingdom; b Biodiscovery Institute, Translational Medical Sciences, School of Medicine, University of Nottingham, Nottingham, United Kingdom; c Department of Eye and Vision Science, University of Liverpool, Liverpool, United Kingdom; d Department of Histopathology, Nottingham University Hospitals NHS, Nottingham, United Kingdom; e Department of Mathematics and Statistics, University of Strathclyde, Glasgow, United Kingdom

**Keywords:** Digital pathology, Tissue microarrays, Stromal stain, Mathematical modelling, Machine learning, Digital multiplex

## Abstract

Whilst automated analysis of immunostains in pathology research has focused predominantly on the epithelial compartment, automated analysis of stains in the stromal compartment is challenging and therefore requires time-consuming pathological input and guidance to adjust to tissue morphometry as perceived by pathologists. This study aimed to develop a robust method to automate stromal stain analyses using 2 of the commonest stromal stains (SMA and desmin) employed in clinical pathology practice as examples. An effective computational method capable of automatically assessing and quantifying tumour-associated stromal stains was developed and applied on cores of colorectal cancer tissue microarrays. The methodology combines both mathematical models and deep learning techniques with the former requiring no training data and the latter as many inputs as possible. The novel mathematical model was used to produce a digital double marker overlay allowing for fast automated digital multiplex analysis of stromal stains. The results show that deep learning methodologies in combination with mathematical modelling allow for an accurate means of quantifying stromal stains whilst also opening up new possibilities of digital multiplex analyses.

## Highlights

• Mathematical modelling and deep learning algorithms can assist analysis of stromal stains in pathology samples including precise digital quantification.
• This process starts with the segmentation of stroma in tissue microarrays to accurately assess stromal stain density within the appropriate stromal area.
• The segmentation method accounts for other structures which may be anatomically included in the stromal area.
• The proposed methodology could be used to aid pathologists and researchers for stain quantification, allowing for precision compared to manual scoring.
• The proposed method has the potential for digital overlap assessment without IHC multiplexing.

## Introduction

The rise of digital pathology and image analysis in recent years has opened up the possibility of semi-automatic and automatic methods to be developed, allowing for relevant immunostains to be detected and inform treatment, diagnosis etc.^1^^,^^2^ Use of both mathematical modelling^3^^,^^4^ (using methods such as variational segmentation and clustering) and deep learning^5^^,^^6^ (using convolutional neural networks (CNNs)) can provide effective pipelines for assessing stains and segmenting regions of interest in microscopy images. The 2 methods are often studied and applied independently due to large differences in how they operate.

Variational methods (mathematical models) typically segment images using models which specify pixels/regions of interest based on analytically defined criteria for example: intensity, shape, smoothness of the region, etc. Though offering an explainable robust framework for segmentation, histological images often need inhomogeneous, irregular regions segmented, and thus applications have been limited.^3^^,^^4^

The introduction of deep learning methods in recent years has helped to improve automated histopathology image analysis beyond previous methods. Deep learning methods have been demonstrated to be more effective than classic machine learning methods in segmenting histological images,^7^^,^^8^ and clustering methods have shown potential to separate tissue micro-environment components like immune cells and cancer-associated fibroblasts.^9^

While the predominant focus of published literature is on epithelial immunostain evaluation,^3^^,^^8^^,^^9^ tumour-associated stromal stain analyses are challenging as the compartment is morphologically complex, including muscles, vessels (small and large), and acellular components which may be stained alongside the stromal cells. As the stromal cells are mainly spindle shaped, there is inherent variability in their size which adds to the complexity of assessment. To accurately assess a stromal stain expression pattern is therefore difficult; yet, given the importance of tumour-associated stroma in terms of the functional biology of tumours,^10^ more and more stromal stains will need to be accurately assessed.

This current study, conceived as a methodology development initiative, addressed both mathematical (variational) models and deep learning (artificial intelligence)-based automated assessment of stromal stains. Alpha-smooth muscle actin (SMA) and desmin, being the 2 most common stromal immunohistochemical stains currently used in a clinical setting, were chosen as exemplars. They signify cellular stromal content and the pathologists’ evaluate skeletal muscle and smooth muscle differentiation, respectively, from these 2 stains. Also functionally, these markers have been found to be expressed in colorectal cancer-associated fibroblasts^11^ or desmoplastic tumoural stroma.^12^ Tissue microarrays (TMAs) are powerful economising tools that allow for the study of multiple tissue samples simultaneously, and it is therefore no surprise that they have been incorporated into digital pathology methodologies, including assessment strategies for prognostication.^13^^,^^14^ In the current study, TMAs stained with SMA and desmin were assessed singly and with dual digital overlay as proof of principle that stromal stains may be efficiently assessed using such techniques on tissue cores. Colorectal cancer (CRC) tissue was chosen as an exemplar, as it poses a significant health burden, being a leading cause of mortality throughout the world, with 1.9 million new cases diagnosed each year and a 5-year survival rate of 50%.^15^ The principal cause of death in patients with CRC is metastasis to the liver or lungs occurring in 25% of patients at diagnosis.^16^ It has been shown that the tumour stroma plays a vital role in the process of epithelial–mesenchymal transition (EMT), a crucial process in invasion and metastasis of CRC. Tumours with high stromal content have been shown to be associated with poor prognosis and stromal stains such as SMA have previously shown their ability to detect cancer-associated fibroblasts (CAFs).^17^ Stromal immunostains may therefore have potential utility in determining patient outcome. This paper therefore addresses the need for novel methods for the automated detection and quantification of stromal stains to serve as an adjunct tool for helping pathologists with their assessments.

## Methods

### Tissue cores and staining

Anonymised tissue cores (in the form of a TMA) were provided from a random cohort of colorectal cancer cases from Nottingham University Hospitals NHS Trust [Ethics approved by Health Research authority, East Midlands - Leicester Central Research Ethics Committee, REC reference: 23/EM/0079; IRAS project ID: 313393]. The tumour cores were selected for each case from 3 different tumoural areas: luminal, central, and peripheral, to account for tumoural heterogeneity. These cores were stained with clinical grade antibodies SMA and desmin and counterstained by haematoxylin [Ventana Benchmark Ultra]. Digital images of SMA and desmin-stained TMA cores were obtained using a DP200 scanner (Roche) (×40 magnification). Digital images of colorectal tissue cores from the Human Protein Atlas (proteinatlas.org)^18^^,^^19^ stained with Vimentin and SMA were also assessed. In total, 6 cores with Vimentin and 12 cores with SMA were available.

### Manual annotations

To inform both methodologies, 113 cores were manually annotated using the hand-guided “Wand-Tool” in QuPath under consultant pathologist guidance. All cores were annotated including their stromal compartments, with care taken to highlight large blood vessels and muscularis if present. This was done as the SMA and desmin stain were specifically assessed in the stroma, excluding confounding staining in these anatomical structures. These annotations formed the basis of both methodologies tested.

### Manual assessment of stain

Where manual assessment of stain was performed, histopathologists used an eye-estimate of the percentage of stromal area stained with SMA and desmin (mentally accounting for area of muscle or large vessels); a stain intesnity of 0, 1, and 2 was allocated on manual judgement. A H-score was produced and median cutoff was generated to classify for “high” or “low” stromal staining. Chi-squared analysis was used to assess significant correlations between manual assessment versus automated assessment as outlined later. Adjusted residuals signify where the significance in tables arise from.

### Stroma region detection by deep learning

The detection of the stromal region was done using deep learning methods as manual annotations were available. Stain segmentation was not achievable via deep learning due to lack of such training data.

#### Deep learning method

**Fig. 1:**
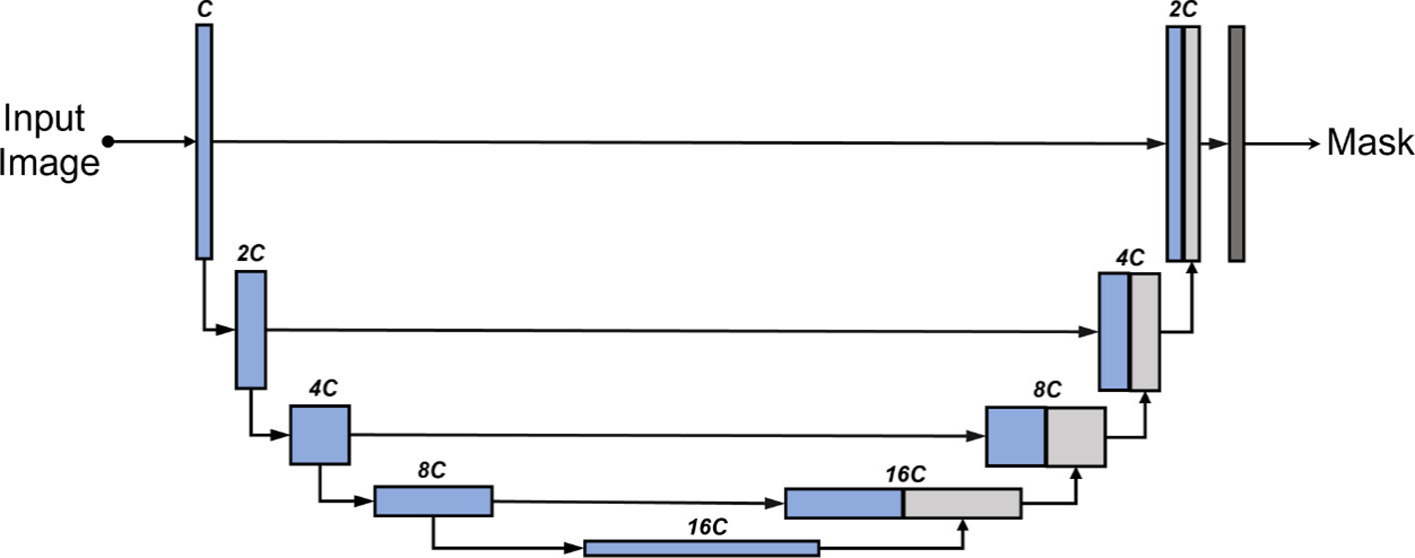
The architecture of U-Net for image segmentation. C represents the number of channels.

A U-Net model^20^ (shown in Fig. 1) was applied for the stromal region segmentation task. Each tissue core was an RGB image, which was then pre-processed and fed into the U-Net. The final layer of the network was a softmax function, with the models output being a predicted mask map ${\left\{U_{k}\right\}}_{k=0,1,\ldots ,K}$, where K is the number of classes. In this case, K=3 and the 3 classes were “background”, “stroma”, and “muscle”. These classes account for acellular stroma, cellular stroma, and muscularis propria/muscle in vessel walls. The involvement of the extra class (i.e., muscle) was under the consideration of the highly visual similarity between stroma and muscle regions. The loss function was the commonly used cross-entropy loss.

For pre-processing, the images of cores cropped from 40× magnification of various sizes were first downsized to 256×256 for both training and testing. Random rotation and flipping operations were adopted on images for data augmentation during training.

### Stain detection by mathematical modelling

For the task of stain segmentation, an unsupervised variational method was used as exhaustive manual annotation of the stained region necessary for a deep learning method was unfeasible. The adopted method is the region-based convex relaxation variant of the Mumford-Shah method, as proposed and studied^21^ for segmentation of multi-channel images. Denote a d-channel colour image as $f=\left(f_{1},\ldots ,f_{d}\right)$ with different colour channels $f_{i}:\Omega \to R$ for i=1,…,d, where $\Omega \subset R^{2}$ is an image domain. The method utilises 3 stages: the convex relaxed Mumford-Shah model in the first stage, lifting the image into a larger image space by combining another colour representation in the second stage, and a thresholding strategy to obtain segmentation in the final stage.

The first stage was achieved by minimising the following functional, as in Xiaohao et al.,^21^ for each channel $f_{i}$ separately to achieve a smooth image $u=\left(u_{1},\ldots ,u_{d}\right)$:

(1) $$E\left(u_{i}\right)=\frac{\lambda }{2}\int_{\Omega }{\left(f_{i}-u_{i}\right)}^{2}\;dx+\frac{\mu }{2}\int_{\Omega }{\left|\nabla u_{i}\right|}^{2}\;dx+\int_{\Omega }|\nabla u_{i}|\;dx.$$

For TMA cores, images are given as RGB and so d=3.

In the second stage, dimension lifting was performed by using the Lab colour space. The 3 channels in the Lab colour space are: perceived lightness (L), green–red colours (a), and blue–yellow colours (b). The Lab space was designed so that a numerical change is proportional to a similar perceived change in colour. It is noted in George^22^ that the Lab colour space is better suited for image segmentation rather than RGB for certain challenging tasks. For the particular case of stain detection in cores stained by SMA and desmin, the stains are coloured brown, and in particular, the b channel in the Lab space segments brown colours well.

**Fig. 2:**
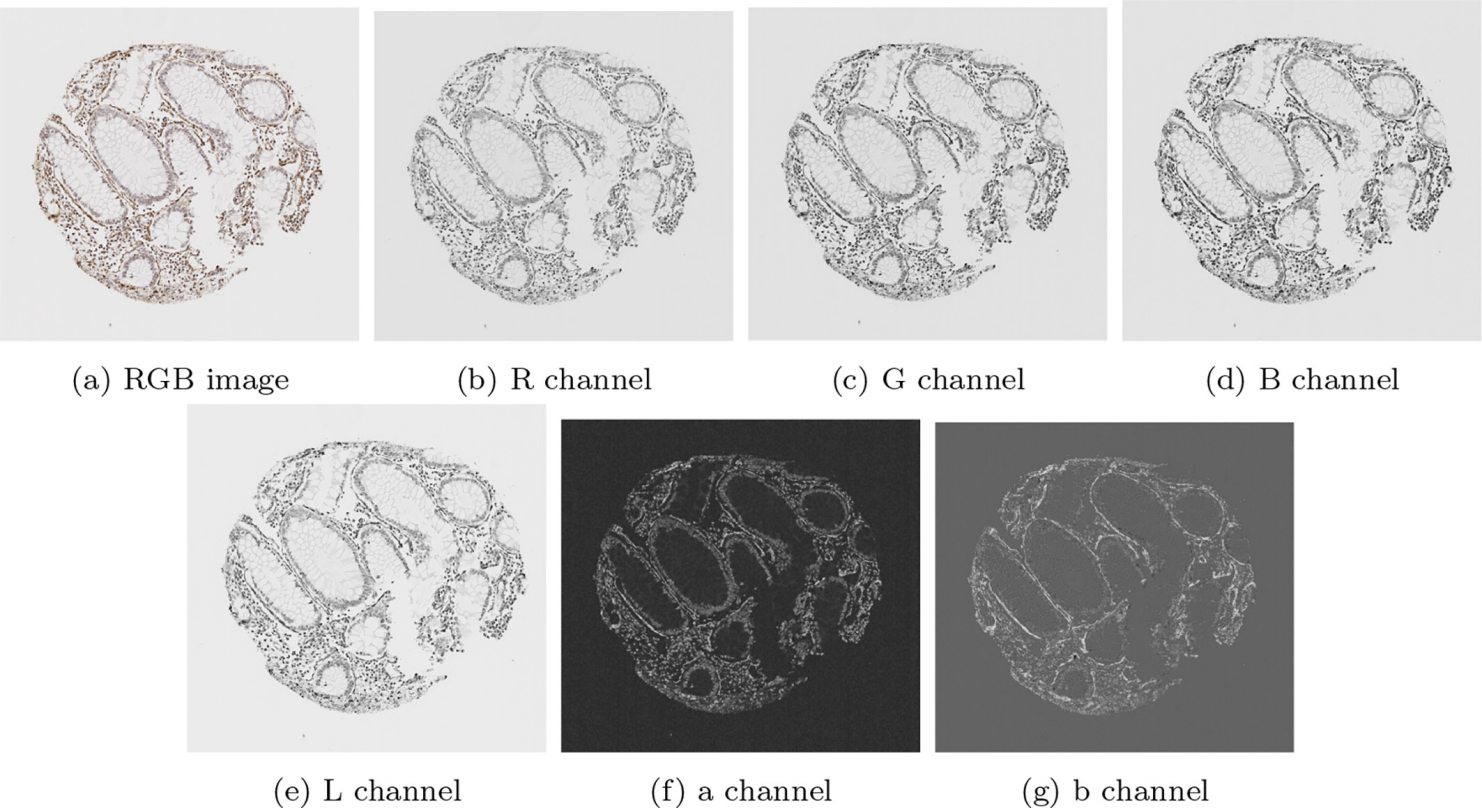
An illustration of each individual channel of an RGB image, and of each individual channel in the transformed Lab space.

The dimension lifting was done simply by concatenating the RGB channels of the restored image u with the transformed image into the Lab space. Let $u\prime =\left({u\prime }_{1},{u\prime }_{2},{u\prime }_{3}\right)$ be the Lab transform of the RGB image u. After concatenation, the vector valued image $u^{*}$ with d=6:

$$u^{*}=\left(u_{1},u_{2},u_{3},{u\prime }_{1},{u\prime }_{2},{u\prime }_{3}\right),$$

is used in the third stage to achieve stain segmentation by thresholding. Fig. 2 shows a typical image of a SMA-stained tumour core and each of the 6 channels utilised.

The third stage achieves segmentation by thresholding. Threshold values can be set manually or found automatically using the k-means algorithm. K-means is an unsupervised clustering method which partitions a set of pixels into K clusters based on their intensities. Similarly, coloured pixels will be grouped into the same cluster.

For this particular application of stain detection, we addressed the challenges arising from the tasks of SMA and desmin stain detection. Threshold values were determined for both cores separately, and in addition, a method was developed which grades the intensity of the SMA staining based on a heatmap. Heatmaps were generated using the intensity of certain channels of $u^{*}$, the output from the variational model. Finally, in the mathematical modelling, an image registration method is applied, which aligns an SMA image with its desmin counterpart, allowing for the region of double staining to be identified.

#### SMA stain detection

While k-means is a popular choice to cluster an image, segmenting the SMA stain using k-means is not sufficient: objects of a similar intensity were also identified in the same cluster as the brown stain (such as muscle, blood vessels, and fibroblasts). Changing the number of clusters in the k-means algorithms hinders performance further, as the staining and similarly coloured unwanted objects were not distinct enough to warrant a new cluster. Therefore, segmentation of the stain only is achieved using 2 steps: an initial k-means run on the whole 6-vector image, followed by a second k-means run on just the b channel in the Lab space.

First, the k-means algorithm is performed on the whole image $u^{*}$, so that the image domain is split into 3 regions, $\Omega =\Omega_{1}\cup \Omega_{2}\cup \Omega_{3}$. The final cluster, $\Omega_{3}$, is a good approximation of the cellular stroma, and as such is later used in results to provide ratios of stained cellular stroma.

However, to achieve stained segmentation only, $\Omega_{3}$ is further refined using the k-means algorithm again with 3 clusters restricted to the domain $\Omega_{3}$, but the image used is the b channel only of the 6-vector image, i.e. ${u\prime }_{3}$. The resulting cluster containing the stain only is defined as $\Omega_{\mathrm{SMA}}.$ A second run of k-means on the b channel is effective at partitioning miscellaneous objects from the stain.

To ensure an automatic method, both $\Omega_{3}$ and $\Omega_{\mathrm{SMA}}$ are taken as the clusters from the respective run of k-means with the largest value in the b channel.

#### Desmin stain detection

For desmin-stained images, the process was slightly different, as the desmin staining is sparse to absent in the cores. The steps included running the variational model (2.5), lifting into Lab space, running k-means on the 6-vector image u. However, the refinement of the k-means result was done by simple thresholding, unlike in the previous SMA case, where k-means was run twice. The k-means algorithm assumes that the size of clusters are of roughly similar, and so k-means would not effective separate the stain from other objects, as the stain is not large enough to be a distinct cluster. Moreover, unlike SMA images, the stain in desmin images is rather distinct, and so, a simple operation like a predefined threshold value is effective. Therefore, the domain containing desmin stain only, $\Omega_{\mathrm{Desmin}}$ is found by thresholding the image in the domain $\Omega_{3}$. Further detail can be found in the results section, in which further development of the model is discussed.

#### Grading SMA stains

As well as assessing the spatial stain distribution, the strength of the stain was also assessed based on the intensity value of the pixels in the segmented stromal area. In SMA images, a darker colour implies a stronger stain while a lighter colour is representative of a fainter stain. A method was developed to class stains into 3 grades by thresholding a heatmap.

To construct the heatmap, the output of the variational model in Lab space was used, but scaled by a factor of 10 in the a and b channels to obtain a vector valued image $w=\left({u\prime }_{1},10{u\prime }_{2},10{u\prime }_{3}\right).$ These 2 channels show a distinction between different shades of brown, allowing for the differentiation of intensity values. Then, the original Lab image $u^{\prime }=\left({u\prime }_{1},{u\prime }_{2},{u\prime }_{3}\right)$ and scaled Lab image w are used as input into the MATLAB function imcolordiff, which calculates the colour difference between images. The output of the function gave H, a heatmap. Most SMA images produced a heatmap with an approximate maximum value of 1.25, though in some cases, a max of 1.4 was noted.

Classifying different grades was done by selecting thresholds applied to H, on the domain $\Omega_{\mathrm{SMA}}$. Denoting the 3 grades as $\Omega_{G_{i}},i=1,2,3$, the 3 sets defining 3 grades were defined as:

(2) $$\Omega_{G_{1}}=\left\{x\in \Omega_{\mathrm{SMA}}:0.35<H\left(x\right)<0.75\right\}$$

(3) $$\Omega_{G_{2}}=\left\{x\in \Omega_{\mathrm{SMA}}:0.75<H\left(x\right)<1.15\right\}$$

(4) $$\Omega_{G_{3}}=\left\{x\in \Omega_{\mathrm{SMA}}:1.15<H\left(x\right)\right\}.$$

#### Alignment of SMA and desmin cores by image registration

With both SMA and desmin cores segmented, the level of double biomarker positive stroma (i.e., the region of the stroma that is stained by both biomarkers) can be assessed. Simple overlaying is not sufficient as the SMA and desmin images are not usually aligned.

Image registration methods are used to align 2 images. The aim of image registration is to find a deformable transformation $y\left(x\right):R^{2}\to R^{2}$ which maps an image T to a fixed image R, with $T,R\in \Omega \subset R^{2}$, such that $T\left(y\left(x\right)\right)\approx R\left(x\right)$. The transformation is usually written as $y\left(x\right)=x+\varphi \left(x\right)$, where $\varphi \left(x\right)=\left(\varphi_{1}\left(x\right),\varphi_{2}\left(x\right)\right)$ is the displacement vector field.

In the case of mapping SMA images to desmin images, the core aim is to map segmented stain from the SMA image using an appropriate map, such that the mapped SMA stain is aligned with the segmented desmin stain. This allows for the comparison of regions where staining is positive for both images. To do this, a variational registration model is implemented, given by the following:

$$\min_{\varphi }D^{\mathrm{MI}}\left(T\left(x+\varphi \left(x\right)\right),R\left(x\right)\right)+\frac{\alpha }{2}\int_{\Omega }\sum_{\ell =1}^{2}{\left|\nabla \varphi_{\ell }\right|}^{2}\;dx,$$

where $D^{\mathrm{MI}}$ is the Mutual Information (MI) similarity measure, Pluim Josien et al.^23^ defined as:

$$D^{\mathrm{MI}}\left(T\left(x+\varphi \left(x\right)\right),R\left(x\right)\right)=-\int_{R^{2}}p_{T,R}\left(t,r\right)\log\frac{p_{T,R}\left(t,r\right)}{p_{T}\left(t\right)p_{R}\left(r\right)}\;\mathrm{dt}\;\mathrm{dr},$$

where $p_{T},p_{R}$ are the probability distribution functions (PDFs) of grey values in T and R, and $p_{T,R}$ is the joint PDF of grey values.

After finding a transformation from the SMA image to the desmin image, assessing the regions of double staining is simple. The transformation was applied to the SMA stain segmentation, and the double-stained region is determined by the intersection of the transformed segmented SMA stain and the segmented desmin stain.

## Results

Combining outputs from both the mathematical model (MM) and the deep learning method (DLM) leads to interesting results, based on their ability to segment out stroma from epithelium (DLM), as well as their ability to identify positive stromal cells (MM) and remove large vessels or muscularis components from the stromal compartment assessment (DLM). In order to identify and segment the stromal region in each TMA core, the DLM is solely utilised due to availability of training data of stromal regions for DLM and MM’s inability to differentiate between cells. The regions within the stromal compartment which had taken up the SMA stain were then identified using the MM due to DLM’s inability to function without training data, and MM’s ability to differentiate contrast in colours within the stromal region, without training data. Finally, the MM was used to identify cases with both SMA and desmin positivity.

### Development of method

Due to the nature of stromal stains, such as SMA or desmin, the complexity of accurately identifying and quantifying the speicfic stromal cell component requires a multi-layered approach. For the segmentation of SMA, running k-means only once would not be sufficient. The difficulty of using the k-means algorithm to partition the SMA images was due to its tendency to cluster the brown staining with unwanted similar colours, such as intense haematoxylin-stained immune cells.

**Fig. 3:**
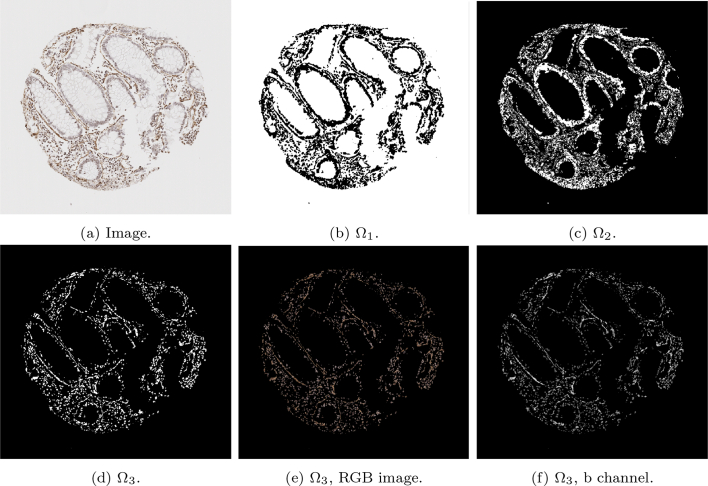
Mathematical output (b)–(d) of the 3 clusters on the given SMA image (a). In addition, (e)–(f) show the RGB image, and the b channel from the Lab colour space of the stain cluster.

**Fig. 4:**
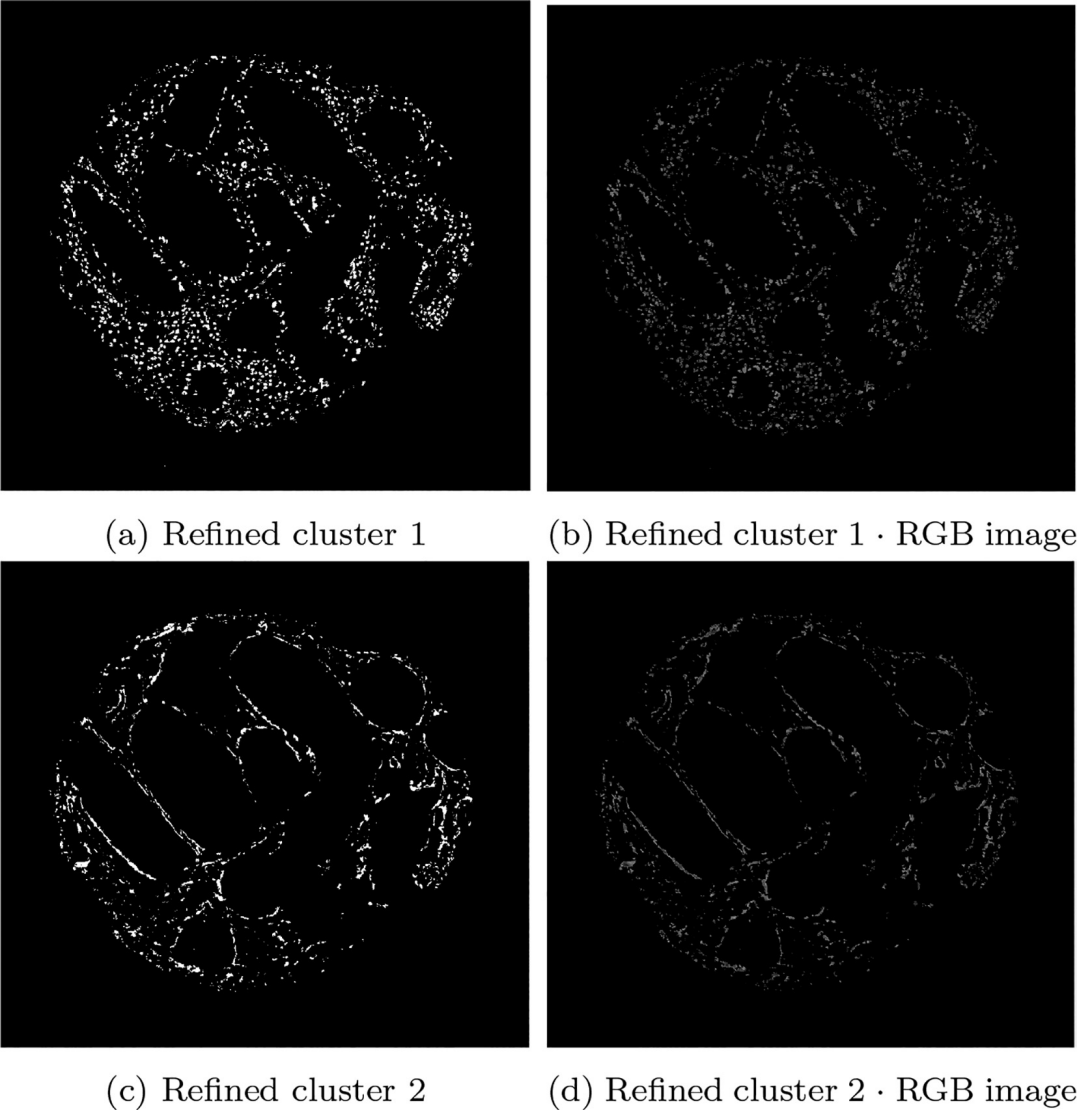
Output of the refined clusters on the image stained by SMA from Fig. 3 (SMA). The top row shows misc objects and the bottom row shows the stain.

An example of the standard k-means output is shown in Fig. 3 on a given SMA image. The image domain is clustered into 3 clusters with the k-means algorithm, such that $\Omega =\cup_{i=1}^{3}\Omega_{i}$. The cluster containing the stain ($\Omega_{3}$) contains additional unwanted objects that differ in colour value only slightly, as shown in Fig. 3e, in which the mask of the cluster multiplied with the RGB image is shown. The difference between the stained pixels and other pixels becomes more obvious when examining the b channel in the Lab space, as shown in Fig. 3f, where the stained pixels have a larger intensity in this channel. Therefore, including a second k-means run on the b channel only allows for the differentiation of the stromal stain from other objects. Fig. 4 shows the refinement of $\Omega_{3}$ from Fig. 3, where the unwanted objects are in one cluster (refined cluster 1) and the stained pixels are in another cluster (refined cluster 2, i.e., $\Omega_{\mathrm{SMA}}$). Note that $\Omega_{3}$ and $\Omega_{\mathrm{SMA}}$ are detected automatically by taking the cluster from the respective run of k-means with the largest value in the b channel.

For desmin stained detection, the k-means algorithm was not suitable to further partition the image domain as k-means assumes the size of each cluster to be relatively equal. Due to the low levels of desmin staining observed in the TMA cores, the required cluster containing the stain would also need to be small to accommodate the level of desmin stain. As a result, the k-means output on desmin images tended to group brown staining with unwanted objects. Moreover, the intensity of staining in desmin images in the b channel of the Lab space is rather distinct, and therefore simple thresholding is a suitable solution to acquire $\Omega_{\mathrm{Desmin}}.$

To formally define this, the stain on the desmin image, $\Omega_{\mathrm{Desmin}}$, is found by thresholding the b channel over the domain $\Omega_{3}$ after an initial k-means run:

$$\Omega_{\mathrm{Desmin}}=\left\{x\in \Omega_{3}:{u\prime }_{3}\left(x\right)>\rho \right\},$$

where ρ=0.65. In principle, the tolerance for choosing ρ for this particular application of desmin staining is wide, as the stains are distinct enough.

**Fig. 5:**
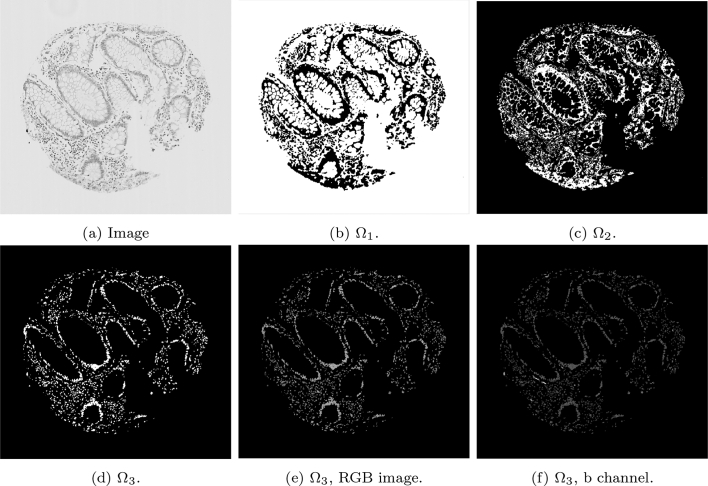
The k-means output (b)–(d) of the 3 clusters on the given desmin image (a), the RGB image of $\Omega_{3}$ (e) and the b channel from the Lab colour space.

**Fig. 6:**
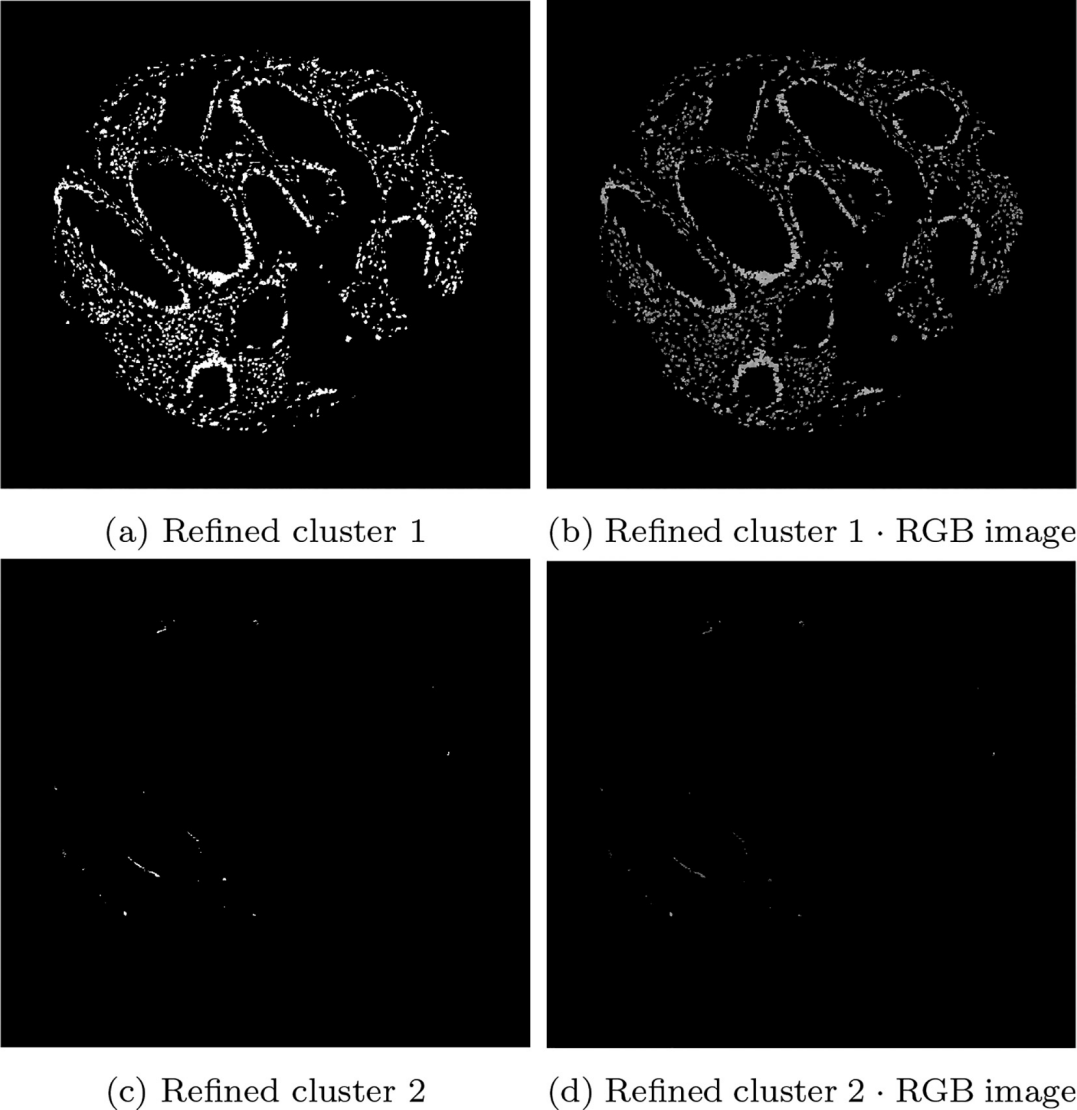
Output of the refined clusters on the image stained by desmin from Fig. 5. The top row shows misc objects and the bottom row shows the stain.

The typical k-means output for desmin images is shown in Fig. 5, in which the cluster containing the stain ($\Omega_{3}$) contained both stained pixels and many unwanted objects. To refine the segmentation, the b channel (displayed in Fig. 5f) is thresholded to obtain $\Omega_{\mathrm{Desmin}}$. An example of refining the initial k-means cluster in this way can be found in Fig. 6.

**Fig. 7:**
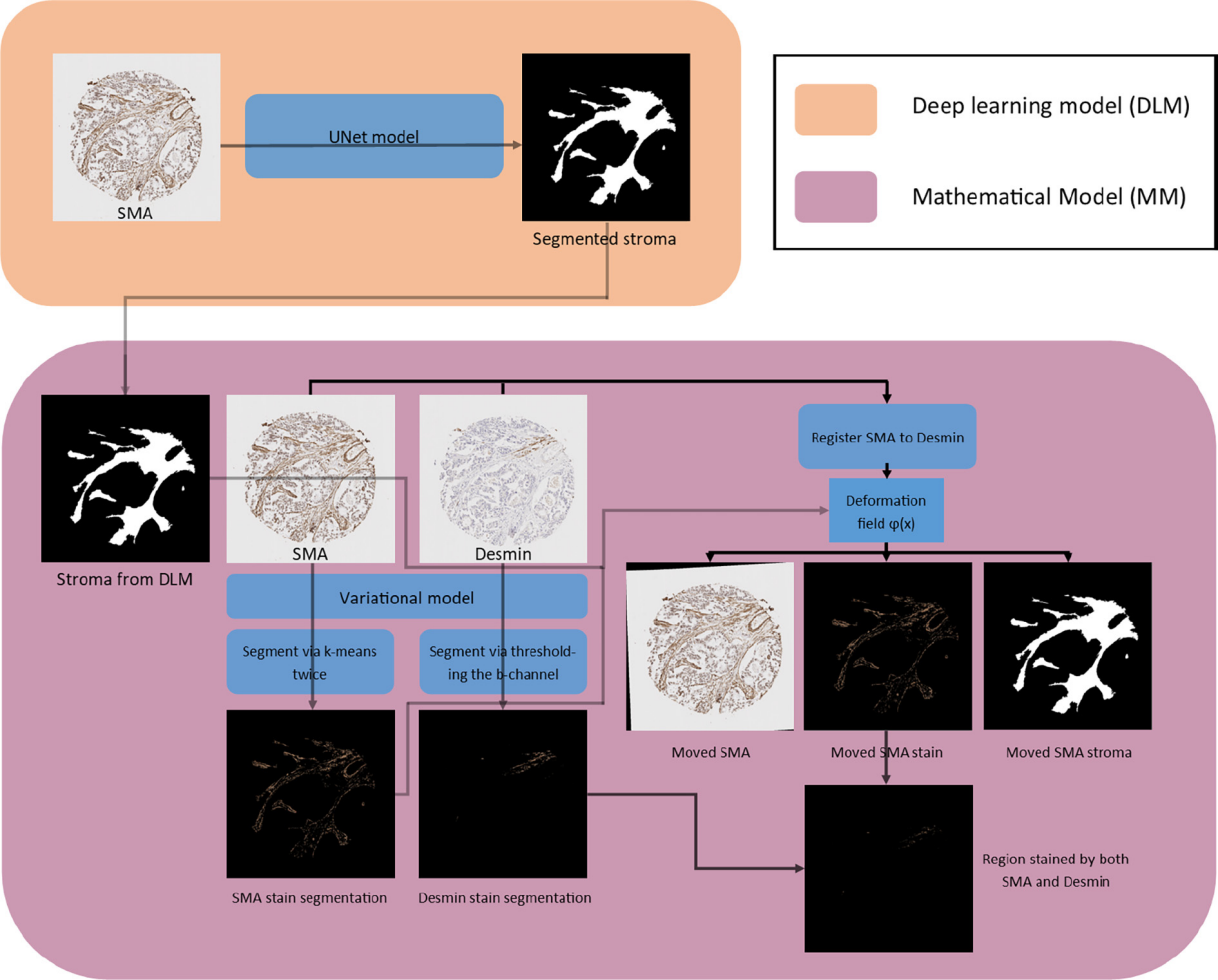
An overview of the mathematical method.

To conclude the development of the methods, a summary of the overall method to achieve segmentation of both the stroma by deep learning and stain segmentation by mathematical model involving: segmentation of both SMA and desmin cores, and alignment of the 2 cores via registration is shown in Fig. 7.

### Analysis of stromal segmentation

Statistical comparisons between average scores for manually annotated stromal segmentation and automated DLM stromal segmentation (cases n=35, cores n=84, a subset of the in-house dataset) are presented in Table 1 with a chi-squared analysis. It was found that the 2 methods were significantly correlated with each other $\left(p\leq 0.001\right)$_,_ whereby both methods would correctly identify cases as either low or high percentage stroma. Only 4 cases differed on final outcome.

**Table 1:** Manual stromal segmentation versus automated stromal segmentation cross-tabulation.

	Automated segmentation		Adjusted residuals		
	Low % stroma	High % stroma	Low % stroma	High % stroma	$\chi^{2}$*P*-value
Manual segmentation					**<.001**
Low % stroma	16 (88.9%)	2 (11.1%)	4.6	−4.6	
High % stroma	2 (11.8%)	15 (88.2%)	−4.6	4.6	

Moreover, 4-fold cross-validation was adopted for the evaluation, where the 113 images were split into a training set and test set with a ratio of 75:25 in each of the 4 independent experiments. Further, 20% of the training set were randomly selected as the validation set. The mean values and standard variances of the corresponding performance metrics are reported in Table 2.

**Table 2:** Mean values and the standard variances of the stroma segmentation performances of DLM. Four-folder cross-validation is adopted. There are 3 categories for the outputs.

Model	Acc	Sens	IoU	Dice
DLM	0.926 ± 0.011	0.822 ± 0.026	0.944 ± 0.007	0.763 ± 0.032

The DLM and manual segmentation of the stromal region were also compared with a trainee histopathologist’s manual scoring of the percentage stroma (cases n=32, cores n=59, a subset of the in-house dataset) presented in Table 3 with a chi-squared analysis. Both methods displayed significant association with the histopathological assessment, however the deep learning method correctly categorised a further 2 cases compared to the manual segmentation method.

**Table 3:** Manual and deep learning stromal segmentation versus histopathologist’s manual assessment cross-tabulation.

	Histopathologist assessment		Adjusted residuals		$\chi^{2}$*P*-value
	Low % stroma	High % stroma	Low % stroma	High % stroma	
Manual segmentation					**.001**
Low % stroma	13 (76.5%)	4 (23.5%)	3.2	−3.2	
High % stroma	3 (20%)	12 (80%)	−3.2	3.2	
Deep learning segmentation					**<.001**
Low % stroma	14 (82.4%)	3 (17.6%)	3.9	−3.9	
High % stroma	2 (13.3%)	13 (86.7%)	−3.9	3.9	

**Fig. 8:**
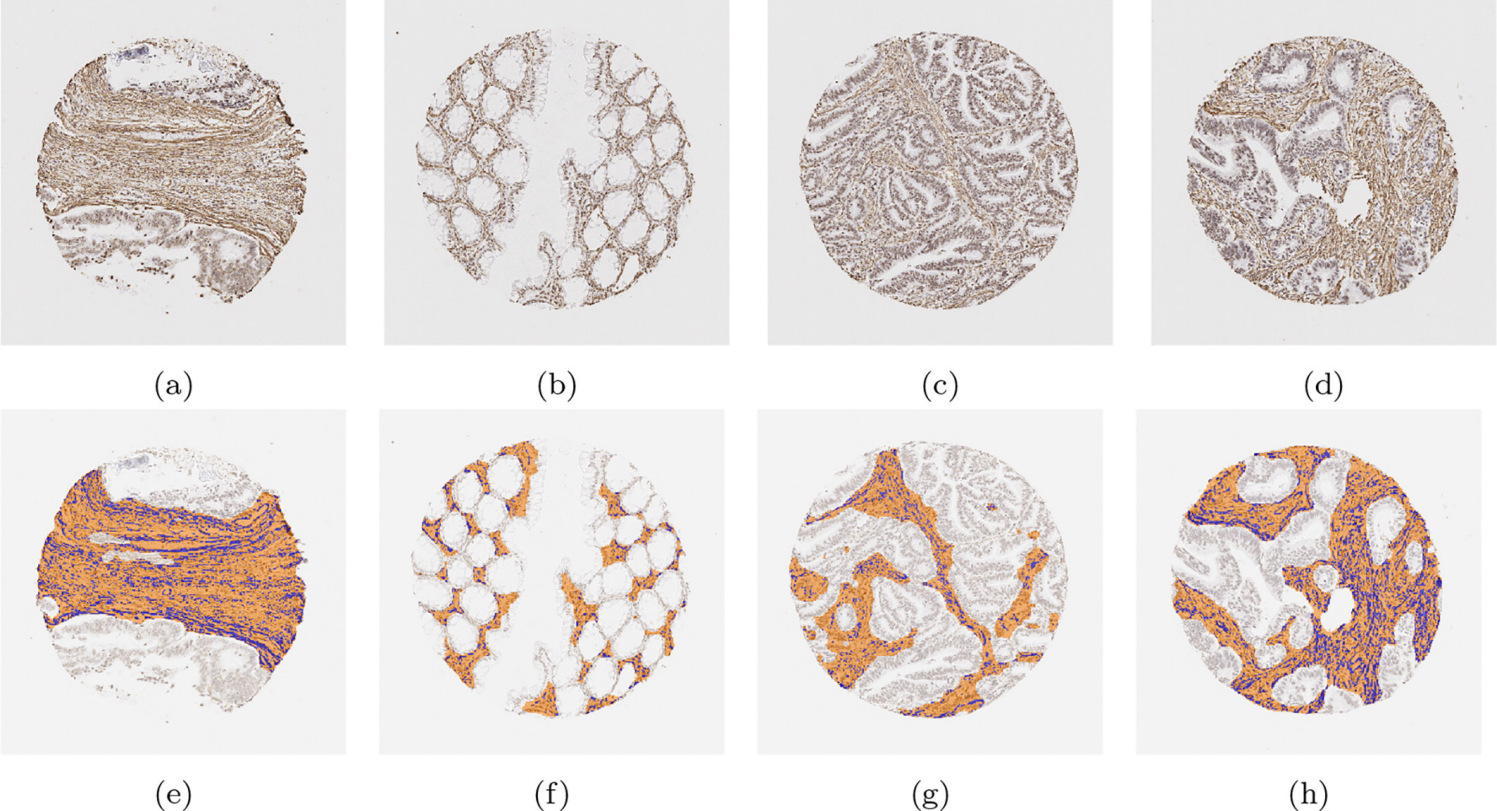
Examples of segmentation results on cores without significant muscle regions. Those in the upper row are the original images, while those in the lower row are the segmentation results. The orange colour represents predicted stroma regions found using the DLM, and the blue colour represents the brown stainings found using the MM.

**Fig. 9:**
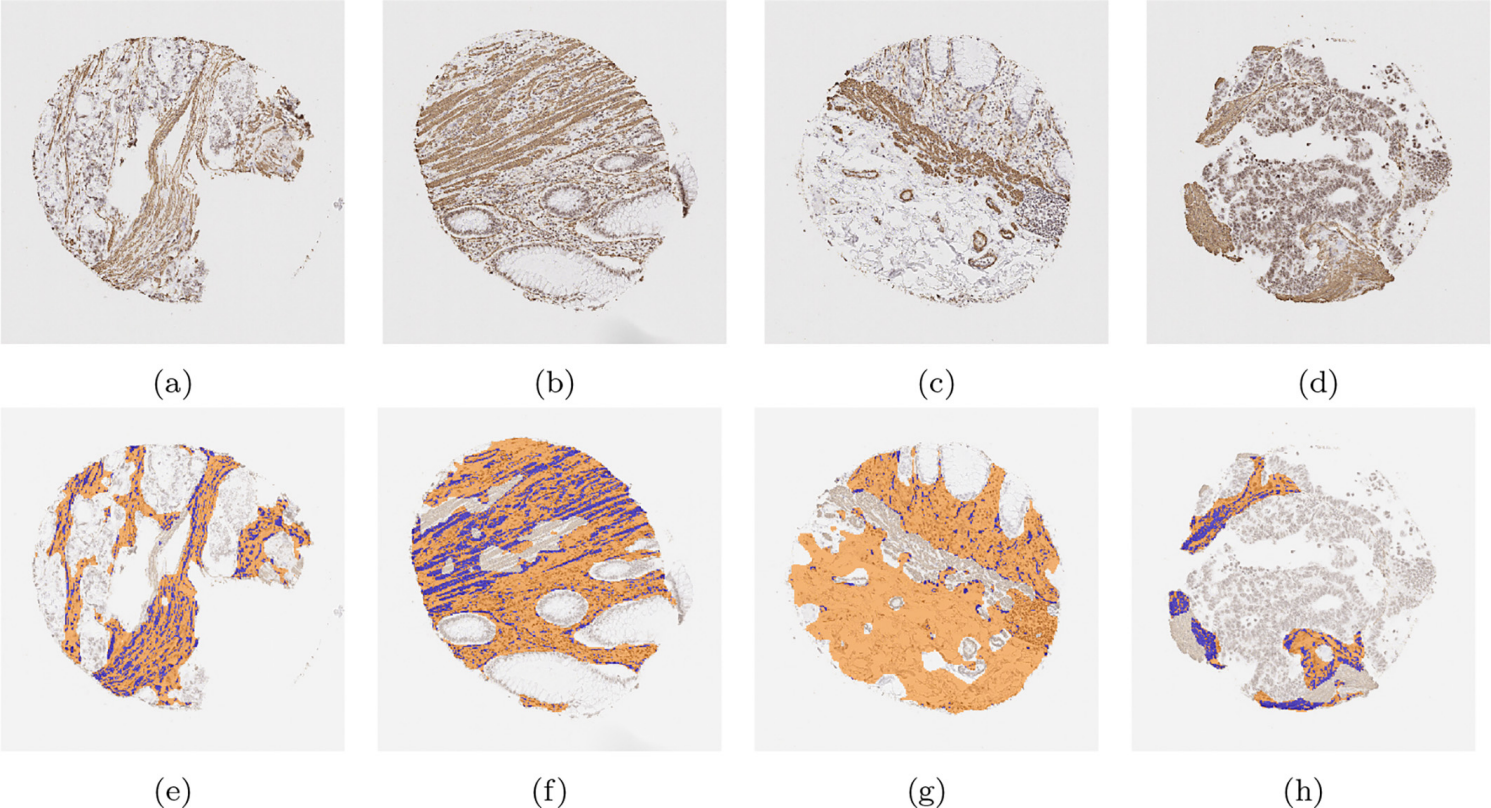
Examples of segmentation results on cores with significant muscle regions. Those in the upper row are the original images, while those in the lower row are the segmentation results. The orange colour represents predicted stroma regions found using the DLM, and the blue colour represents the brown stainings found using the MM.

To clearly determine stromal stain expression, the DLM was designed to exclude regions of muscle and large blood vessels where possible. Fig. 8 is representative of no muscle regions in the cores, whilst Fig. 9 is representative of substantially large muscularis regions in the cores. It is observed that the trained model can recognise the stromal regions with high accuracy. However, as seen from Fig. 9, in some difficult cases, the model still had a tendency to mistakenly recognise muscle regions as stromal regions. This is mainly due to the high morphological similarity between stromal cells and muscles fibres.

### Analysis of SMA stained stroma

**Fig. 10:**
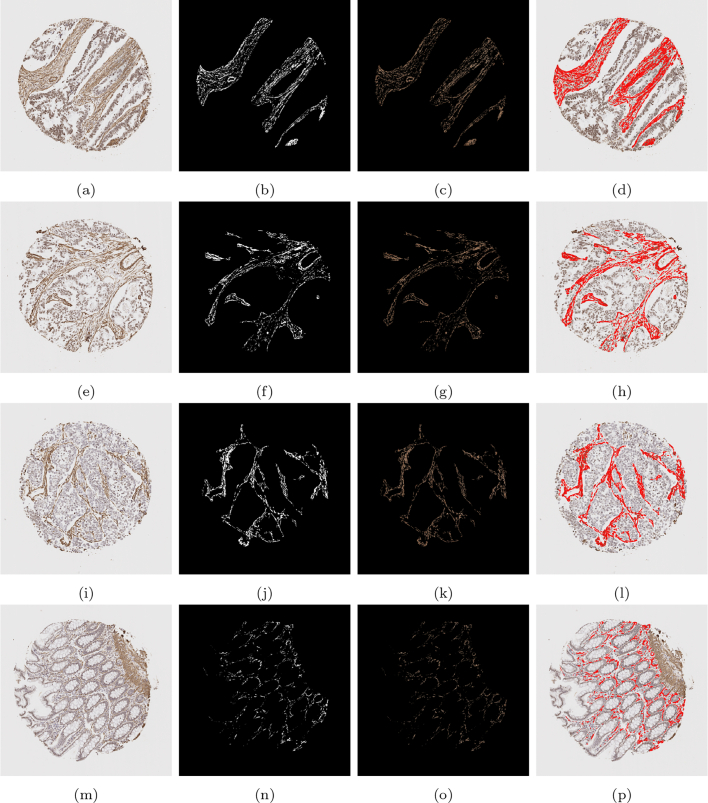
A compilation of results from the mathematical model detecting SMA staining. The first column shows the original image, the second column shows the binary segmented stain, the third column shows the segmented stain in RGB, and the final column shows the segmented stain overlaid on the original image.

Using the SMA segmentation result, 2 scores were produced: The first score, denoted as SMA 1, was the percentage of stain taken up with respect to the area of the entire stromal compartment. This stromal compartment score is segmented using DLM, and the stained region segmented and quantified using the MM. The second score, denoted as SMA 2, is a percentage of stain taken up with respect to the area of the cellular stroma, which is more likened to the way in which a histopathologist would quantify a stromal stain. The cellular stroma region is taken as $\Omega_{3}$, as defined in Section 2.5.1, which is the initial output of the k-means algorithm before refinement. Some examples can be found in Fig. 10, and quantitative results for the SMA scoring using these 2 methods can be found in the first and second column of Table 4, respectively.

**Table 4:** Quantitative scores for cores shown in Fig. 10, Fig. 11, Fig. 12, Fig. 13. Including the first method of SMA scoring, negative scores as well as the 4 intensity grades of SMA stromal stain (G0, G1, G2, and G3), the associated H-Score, the second method of SMA scoring, the desmin score, and the double positive score.

	SMA 1	SMA 2	G0	G1	G2	G3	H-Score	Desmin	Double
Case 1	23.8	67.4	32.6	7.91	59.1	0.386	127.3	0.03	0.02
Case 2	19.2	67.9	32.1	2.38	62.0	3.52	136.9	2.35	0.65
Case 3	32.2	83.3	16.7	20.6	62.1	0.647	146.7	0.12	0.04
Case 4	8.0	33.4	66.6	7.17	25.7	0.499	60.0	0.13	0.02

**Fig. 11:**
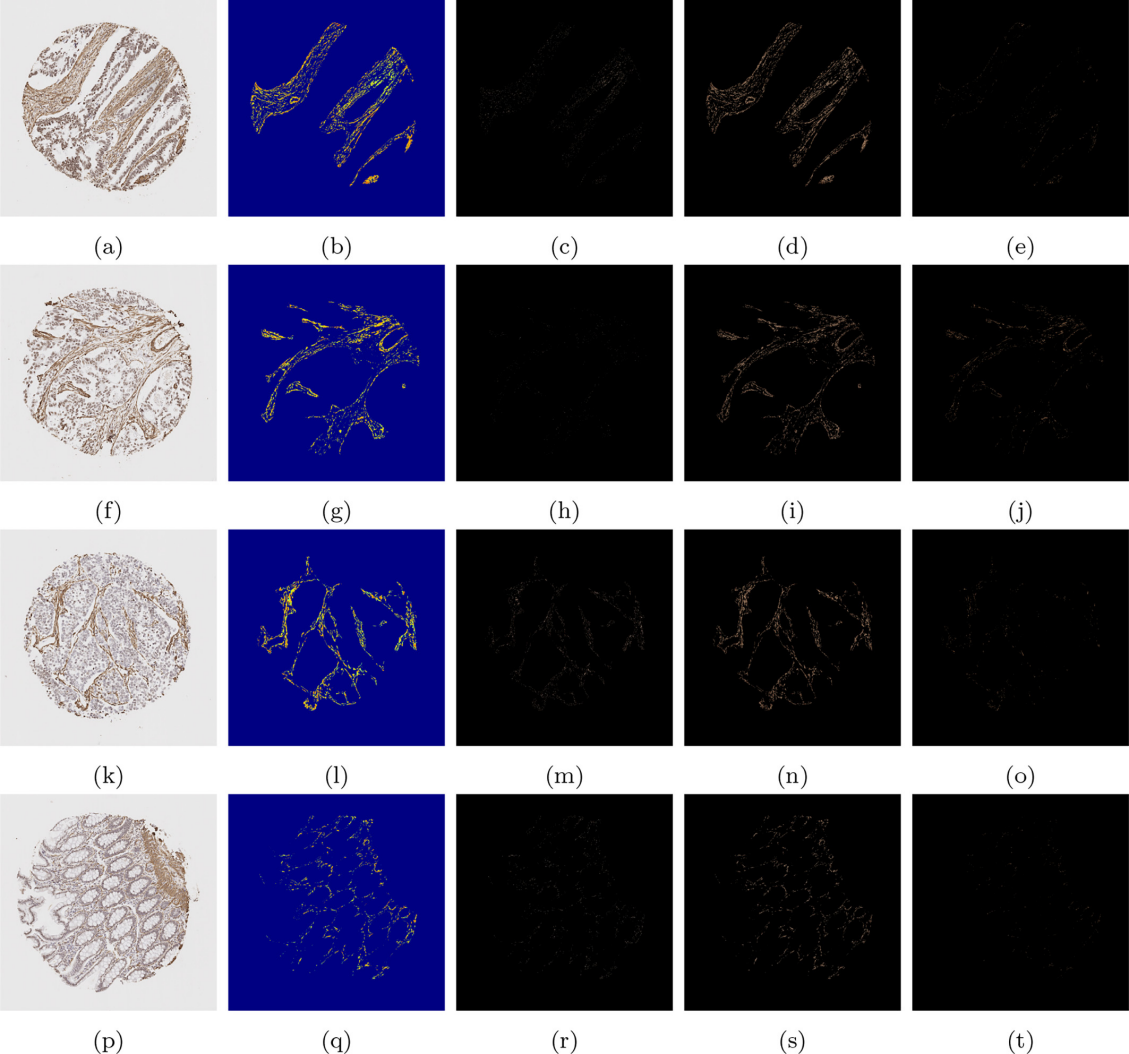
Heatmaps from the images shown in Fig. 10. The first column displays the original image, the second column shows the resulting heatmap, and the third, fourth, and fifth columns show the stain designated as grades 1, 2, and 3, respectively.

Additionally, in Fig. 11, the associated heatmaps generated by the MM are shown, allowing for the classification of the stain into intensity grades 1–3. This allows for an image-based H-score to be calculated, capturing both the intensity and the percentage of SMA/desmin positivity from the segmented stromal cells. Thus, offering an effective automated means to accurately quantify stromal biomarker expression. The scores for intensities 1–3 (as well as the negative area denoted as intensity 0) are shown in Table 4, in which the number reflects the percentage of stromal stain categorised into the respective grade with specifically for the area of the stromal cells within the stromal compartment. Subsequently, the image analysis based H-Score is calculated and displayed in the following column.

### Analysis of desmin-stained stroma

**Fig. 12:**
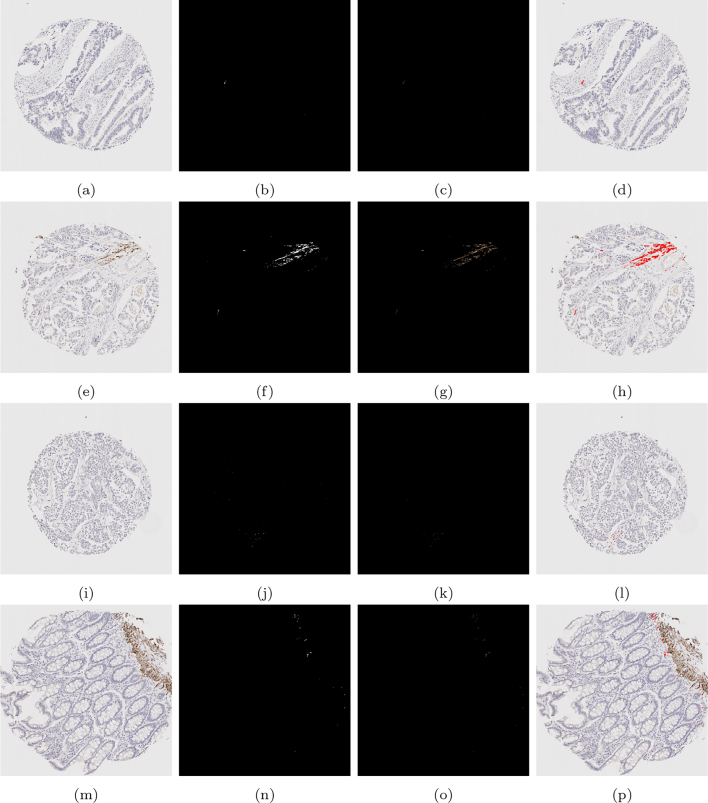
A compilation of results from the mathematical model detecting desmin staining. The first column shows the original image, the second column shows the binary segmented stain, the third column shows the segmented stain in RGB, and the final column shows the segmented stain overlaid on the original image.

Quantitative scoring on the cores stained by desmin is done by the MM only. Fig. 12 shows some examples on some cores. Quantitative results for the scoring of these cores can be found in the second last column of Table 4. This score is a percentage of stain taken up with respect to the area of the entire stromal compartment, defined exactly the same as the first SMA score. In order to generate this score, the stromal region of the core must be found. To acquire this, the SMA core is registered to the desmin core, and the DLM stromal segmentation output is also registered to provide the stromal segmentation of the desmin core.

### Overlay of SMA and desmin-positive stroma

**Fig. 13:**
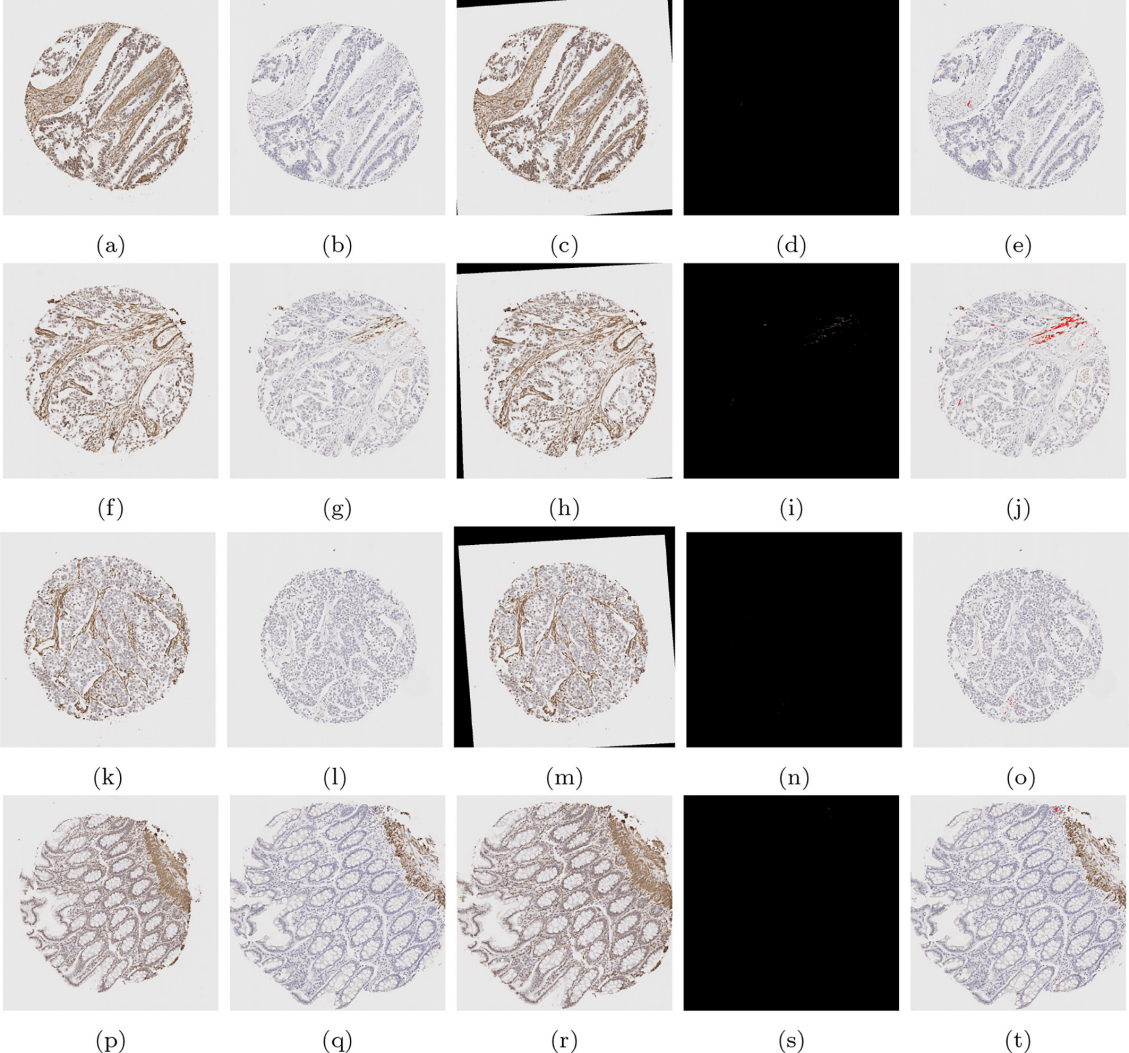
A compilation of double stain analysis. Results for SMA and desmin stain segmentation can be found in Fig. 10, Fig. 12 respectively. In the first column, the SMA image is shown, in the second column, the desmin image is shown, and in the third column, the registered SMA image to be aligned with the desmin image is displayed. In column 4, the binary region of double staining is displayed, and in the final column, the double stained region is overlaid onto the original desmin image.

Analysing the region of double positivity requires first alignment of the SMA image to desmin image, as in general, the 2 images do not coincide with each other. As described in Section 2.5.4, this is achieved by registering the SMA image to the desmin image. Some sample results are displayed in Fig. 13, in which the SMA and desmin images are displayed in the first 2 columns, and the registered SMA image is shown in the third column. With the same transformation, the segmented SMA stain (as shown in Fig. 10) is moved and displayed where it coincides with the desmin stain (as shown in Fig. 12), which is displayed in the final 2 columns. Quantitative results for the scoring of these cores can be found in the final column of Table 4. This is a score of the percentage of stain taken up in both cores with respect to the entire stromal compartment.

### Bland–Altman

**Fig. 14:**
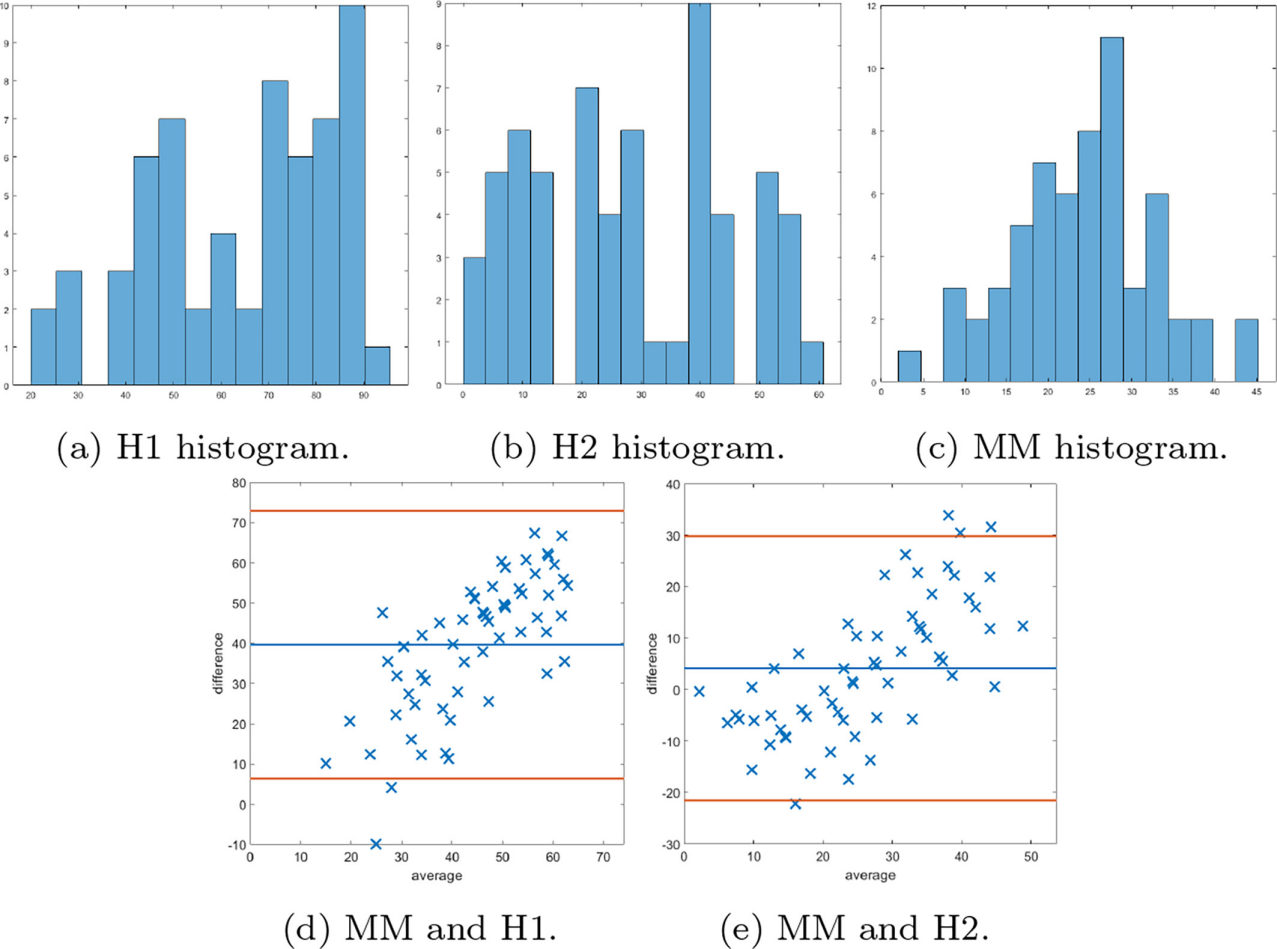
The first row shows the histogram of the scores provided by: histopathologist 1 (H1), histopathologist 2 (H2), and the MM. In the second row, Bland–Altman plots display the discrepancy in SMA scoring comparing the 2 methods with scoring H1 and H2. The MM correlates more with H2.

**Fig. 15:**
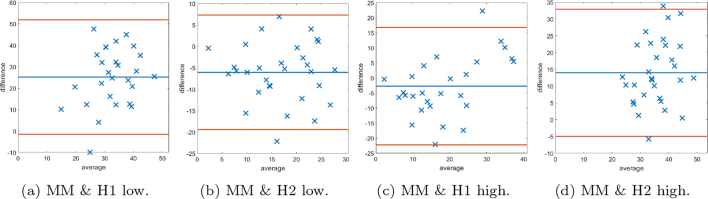
Bland–Altman plots of SMA scoring based on splitting the data into “low” and “high” according to the median cutoff of the respective histopathologists’ scores. Examples of cases scored as “low” by the respective histopathologist on the left, and similarly on the right display plots scored as “high”.

**Fig. 16:**
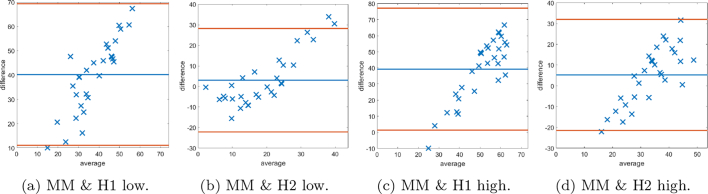
Bland–Altman plots of SMA scoring based on splitting the data into “low” and “high” according to the median cutoff of the MM method. On the left examples of cases scored as “low” by the MM, and similarly on the right display plots scored as “high”.

**Fig. 17:**
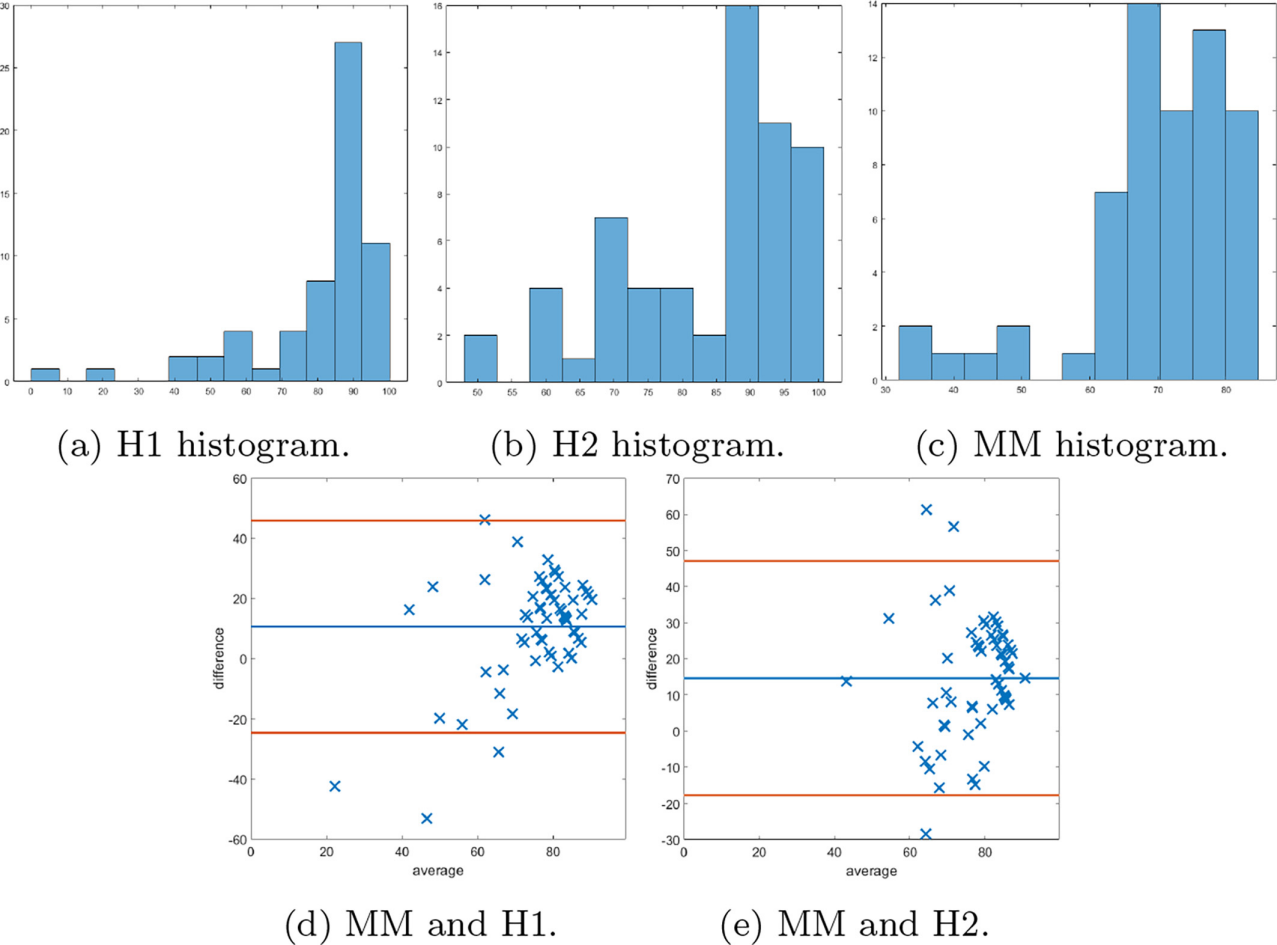
The first row shows the histogram of the scores provided by: histopathologist 1 (H1), histopathologist 2 (H2), and the MM for SMA staining as a percentage of the stromal cells. In the second row, Bland–Altman plots display the discrepancy in SMA scoring comparing the MM with scoring H1 and H2.

**Fig. 18:**
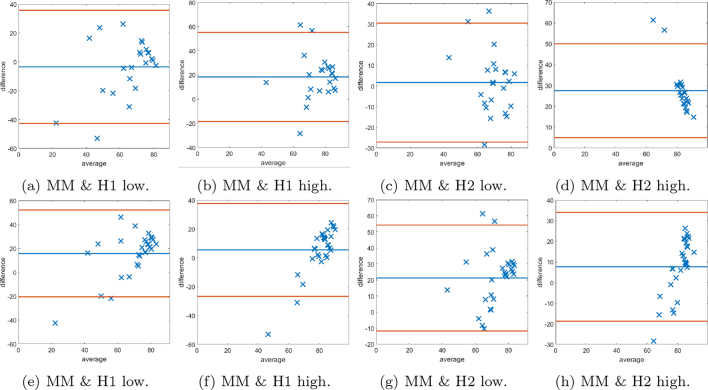
The first row shows Bland–Altman plots of SMA scoring stromal cells based on splitting the data into “low” and “high” according to the median cutoff of the respective histopathologist. The second row shows Bland–Altman plots of SMA scoring on stromal cells based on splitting the data into “low” and “high” according to the median cutoff of the MM.

**Fig. 19:**
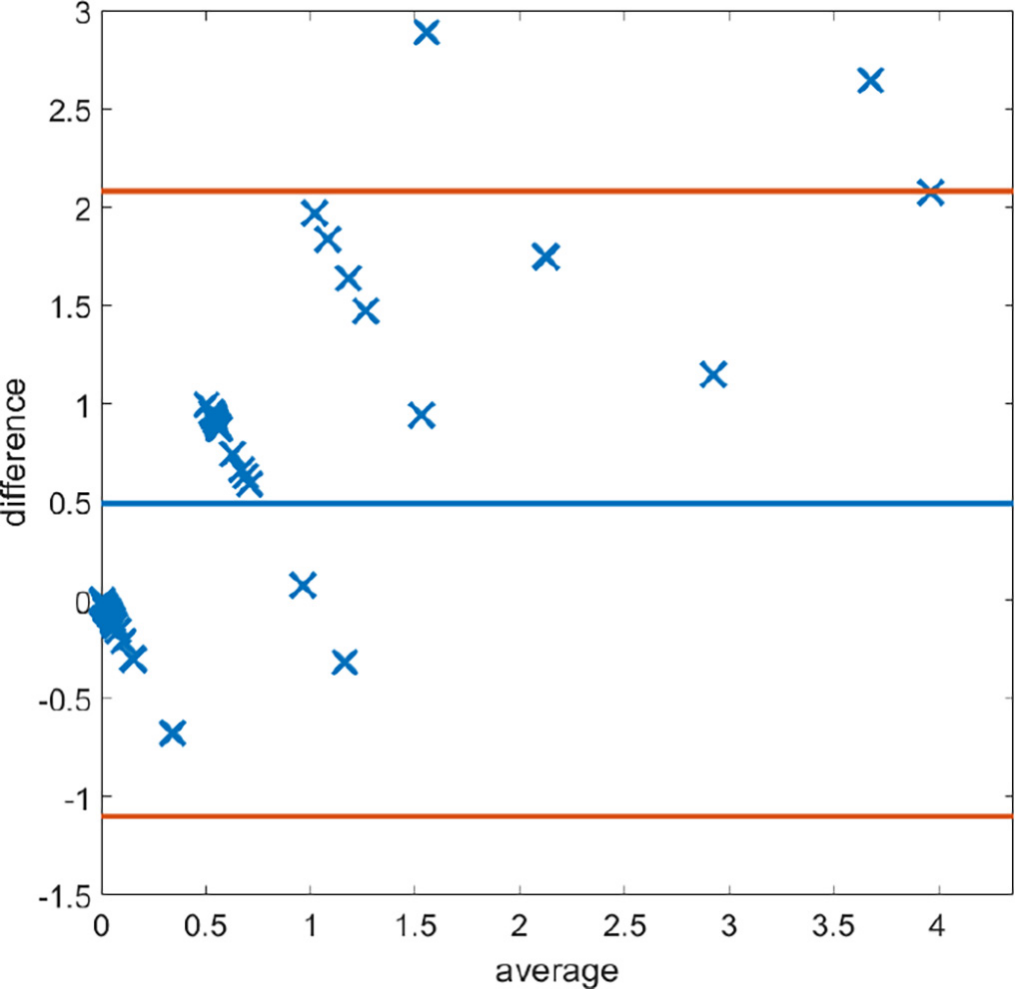
Bland–Altman plots displaying discrepancy in desmin scoring between the mathematical model and histopathologist 1.

Bland–Altman plots are provided to demonstrate the differences between scores provided by the MM and 2 histopathologists. Plots comparing both methods of SMA scores are given (firstly, as a percentage of the stromal compartment and secondly, as a percentage of stromal cells). In Fig. 14, the Bland–Altman plots for the first method of scoring SMA cores are shown, as well as show the histogram of the scores. It is noted that the manual scores by the histopathologists have a tendency to underestimate “low” scores and overestimate “high” scores, and therefore further Bland–Altman plots are constructed in Fig. 15, Fig. 16, in which the data has been split into “low” and “high” according to the median cutoff of the histopathologists’ scores in Fig. 15, and the median cutoff of the MM method in Fig. 16. Similar plots for the second method of scoring SMA cores are shown in Fig. 17, Fig. 18. Finally, the Bland–Altman plot for scoring on desmin cores is shown in Fig. 19.

### Human Protein Atlas

**Fig. 20:**
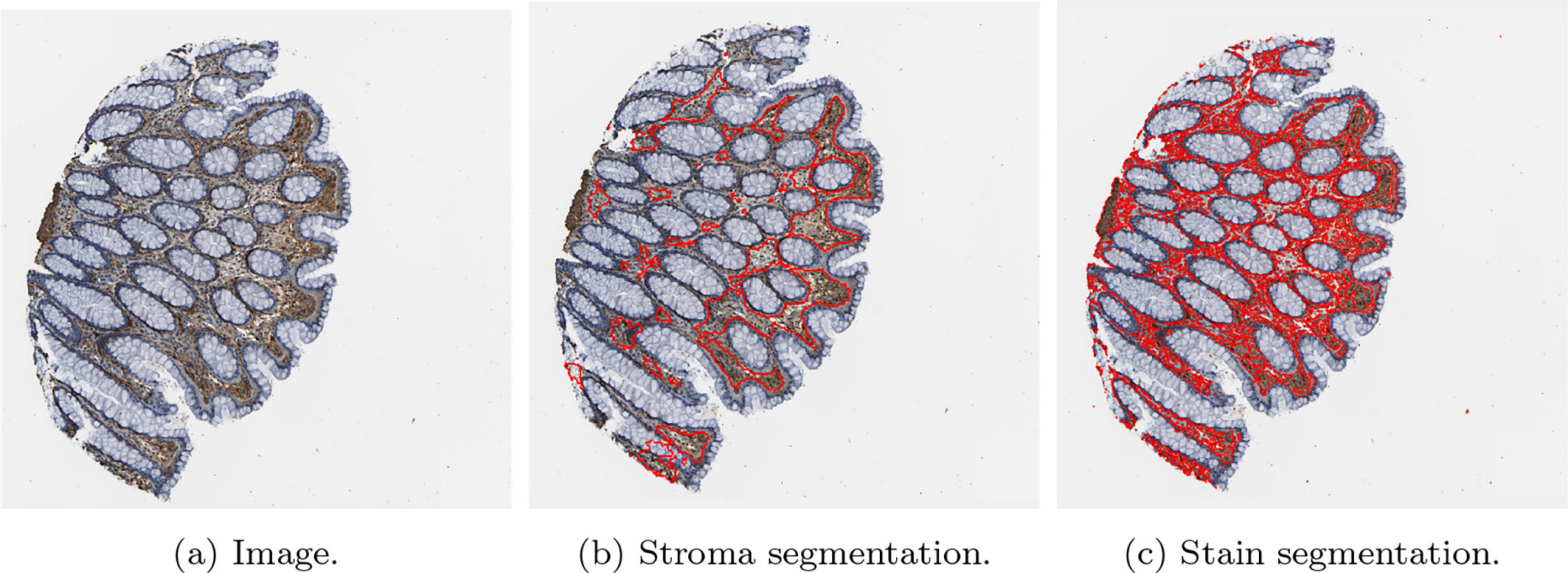
An example of a result of CRC tissue stained with SMA, (https://www.proteinatlas.org/ENSG00000107796-ACTA2/tissue/colon) from the Human Protein Atlas.^19^

**Fig. 21:**
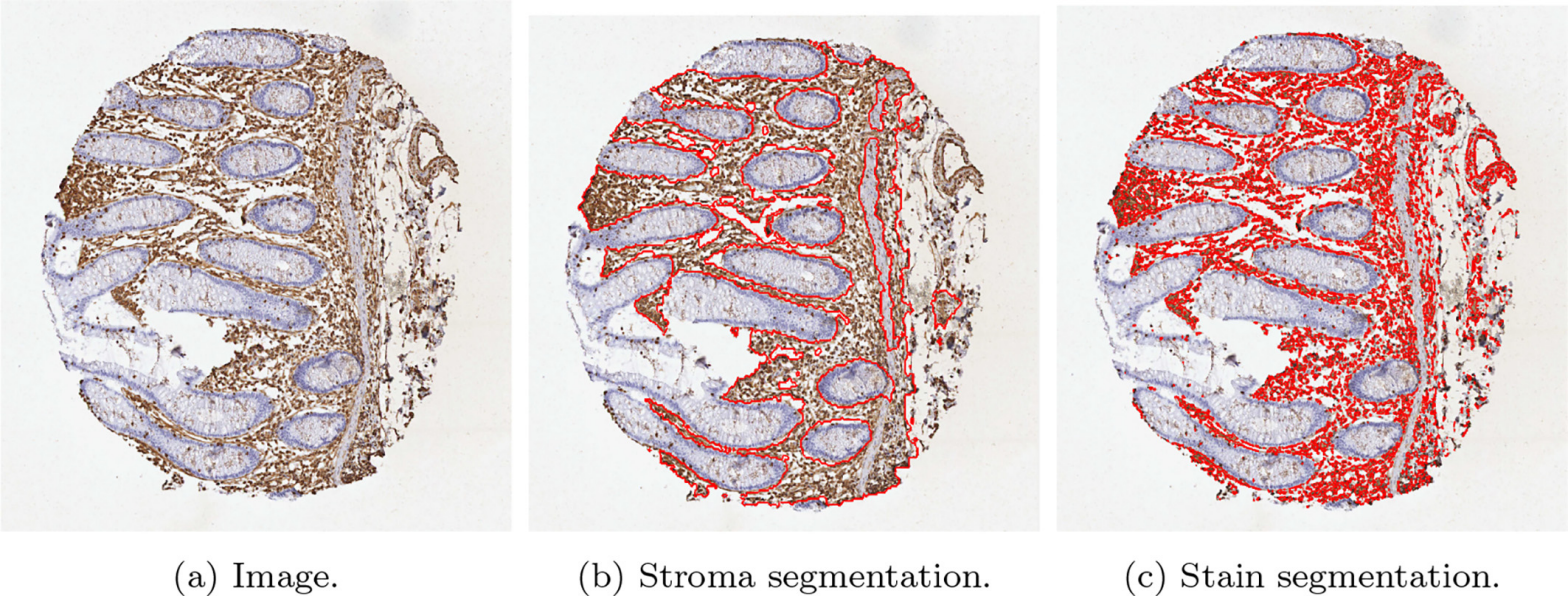
An example of a result of CRC tissue stained with vimentin, (https://www.proteinatlas.org/ENSG00000026025-VIM/tissue/colon) from the Human Protein Atlas.^19^

**Fig. 22:**
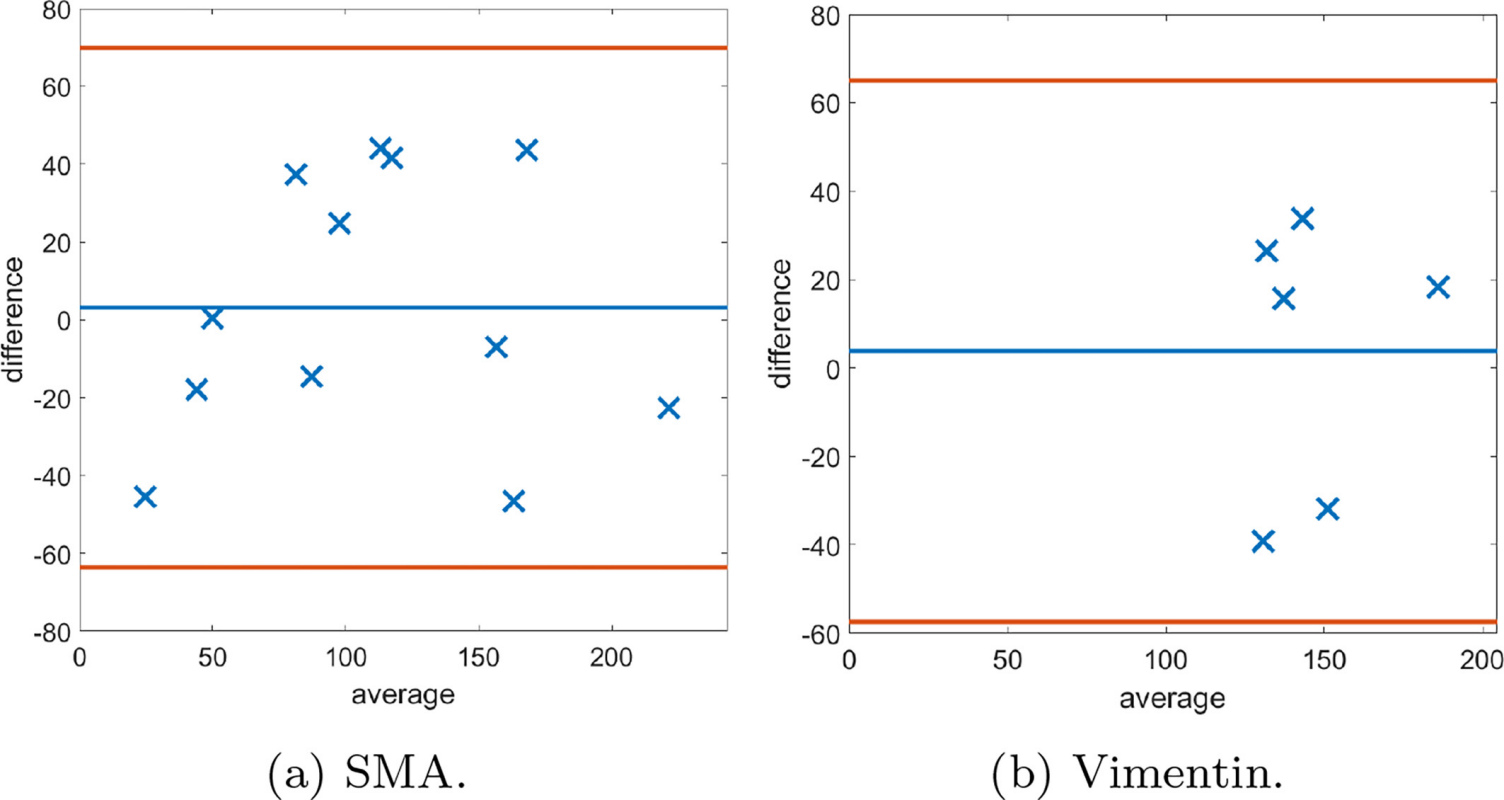
Bland–Altman plots of H-Scores on the Human Protein Atlas images^19^ of both SMA and Vimentin.

The proposed method was applied to CRC TMA images from the Human Protein Atlas.^18^^,^^19^ Two independent stromal stains were analysed: 12 cores stained by SMA and 6 cores stained by vimentin. Cores were assessed digitally using the MM and manually by a histopathologist. Assessment of stains from manual assessment and proposed model correlate well, though correlation of stroal detection was not as strong. Some example stain segmentations of SMA are shown in Fig. 20 and of vimentin in Fig. 21. A Bland–Altman plot of the H-Score comparison is shown in Fig. 22.

## Discussion

In histopathology practice, in contrast to epithelial stains, which are relatively easier to annotate and asssess both manually and digitally, stromal stains present unique challenges. These may be due to stromal composition, cellular or acellular; stromal cell irregular morphology or apparent interdigitation/overlap. This study therefore aimed to develop an efficient automated flowthrough to analyse stromal staining which abrogate the aformentioned challenges. The holistic approach involved both assessment of the percentage stromal area as well as the percentage of stromal cells that were stained. Confounding staining in smooth muscle fibres of the muscular bowel wall and muscular vessel walls was accounted for.

Two approaches for the automated determination and quantification of stromal stains within CRC were developed. In order to determine whether the 2 methods were comparable to the current clinical standard assessment, 2 histopathologists (1 at an early training stage (HP1) and 1 a specialist (HP2) examined the stained cores.

The DLM tackled the problem of stromal detection, and was found to be successful at determining high and low percentage stroma comparable with the pathologists’ manual estimation. Knowing the methods were comparable to clinical estimations of percentage stroma, quantification of stromal stains followed using the MM, which relies on the segmented stromal compartment from the DLM, was similarly comparable to manual scoring, as corroborated by the chi-squared tests. As demonstrated in the Bland–Altman plots, of particular interest is the potential unconscious bias of scoring to extremes from the histopathologists’ scores. It is noted that there may be a tendency to underestimate scores with low staining, and overestimate cores with high staining, as depicted by the histograms of each histopathologist. This is an advantage of the MM, as it is objective and has inherent quantitative accuracy.

The SMA scores obtained by the MM discussed so far are representative of the area taken up by the stain with respect to the entire stromal compartment, cellular and acellular. The MM is also capable of giving a score of the area stained with respect to the cellular stroma only. This latter method is representative of how a stromal stain such as SMA would be quantified to help clinicians and avoid over- or underestimation. The struggle to mentally account for acellular components may also be relieved.

In contrast to SMA stain which has a wider natural range of variability between tumours, desmin, as a stromal stain in colorectal cancer, is skewed towards the underexpressed range. Here also, the MM proves more accurate and inherently outperforms manual scorers in the sub 1% range. Quantification via pixel intensity is therefore likely to be more accurate in quantifying scantly stained stromal stains at relatively fast speeds.

In the rapidly transforming field of digital pathology, segmentation of epithelium and stroma have been attempted by several studies using mathematical modelling, including a level-based active contour method and clustering.^4^ However, use of a simple piecewise-constant intensity distribution assumption is likely to lead to poor performance on complex images. Other researchers^3^ were able to differentiate between tumour and non-tumour epithelium in WSIs of CRC by training a self-organising map. This approach was effective, but if it needs to be colour-independent, as when assessing stromal stains, it would perhaps struggle at quantification.

In contrast to previous mathematical models, the MM presented in this paper is novel in that it was primarily built to assess the staining in the stromal compartment as opposed to concentrating on epithelial stains. As the starting point to the analysis, the use of a relaxed Mumford-Shah variational model allows for piecewise-smooth intensity distributions, meaning a more sophisticated segmentation output is produced when compared with a piecewise-constant assumption. Moreover, the MM method utilises the 6 colour channels jointly and separately where appropriate, improving on results where colour channels are treated individually.

The methods used in this paper avoid pre-selecting tumour regions before algorithm application and/or selecting a stromal hotspot.^24^^,^^25^

In contrast to the proposed method, the aforementioned studies all require user interaction when processing new unseen data, and are therefore not fully automated. In particular, the DLM is able to provide a segmentation of tumour and stromal regions on SMA-stained TMA cores automatically without any user interaction. Tumour and stromal regions are also segmented on desmin images using the same DLM by registering the 2 cores together, thereby moving the stromal region segmented on the SMA image to the desmin image.

While there exist automated methods of tumour epithelium segmentation,^3^^,^^14^^,^^26^^,^^27^ these works typically focus on the sole task of segmentation rather than quantifying immunostain expression. The main novelty of the proposed work is in its ability to both segment out the stromal compartment, and to produce multiple H-scores for multiple stromal stains without the need to alter the DLM. With the stromal and epithelium regions detected by the DLM, and the stained regions detected by the MM, the proposed method can produce scores for the ratio of stained stroma with respect to the total stromal area. The ability of the MM to automatically categorise regions into grades based on the intensity of the stain, providing an image-based H-score, parallels commonplace methods like H-scores done by practicing pathologists, with the advantage of providing objectivity in assessment. Further to this, the MM is also able to detect only the stromal cells within the region, which gives the ability for the proposed method to produce a score for the ratio of stained stroma with respect to the area of the stromal cells. While the presented results achieve this on SMA and desmin staining, there is no limit to the number of stromal stains this method could quantify at a single time. With minor alterations to the clustering method to account for the differences in stromal stain expression, it would be possible to detect the stained regions with very similar methods.

Due to the complexity of multiple pathways involved in cancer, multiple factors contribute to cancer progression. IHC multiplexing has been an innovative tool to extrapolate data regarding several protein interactions within tissues, however this is an intricate and often costly process. Therefore, the ability to carry out digital multiplexing of stromal stains is extremely desirable. To the best of our knowledge, the use of registration techniques to align multiple versions of a TMA slide to facilitate a digital multiplexing method have not been published. Aligning 2 TMA slides via registration, using a rigid transformation with normalised cross-correlation as the similarity measure has been attempted previously,^28^ but did not incorporate the additional information of the stain segmentation as in the proposed method. It is therefore an advantage of the MM that precise regions of double staining can be detected.

The proposed registration method seems to be rather robust in aligning the 2 images, which allows for accurate merging of the 2 stained segmentations. This would prove extremely useful in novel immunohistochemical studies to help elucidate pathways and perhaps produce tools for multi-overlay image panels to help predict operative functional pathways in tumours, which in the long run, could alter patient stratification and prognosis.

To provide further validation of the methodology, we applied the MM to open access images of CRC cores stained with SMA and vimentin, with vimentin being another mesenchymal stain used commonly in clinical practice. The results highlighted the ability of the model to function regardless of a users’ staining protocol or the reagents used to stain tissue. This is often a pitfall of many automated quantification methods as changes in staining intensities lead to discrepancies between results, and may inhibit the algorithm from working correctly. The accurate quantification of a third party core stained with vimentin also emphasises the methodologies’ ability to segment and quantify any stromal stain. However, a potential limitation of the proposed workflow is that a limited set of data was available for training, and so tuning the DLM was infeasible. Therefore, a larger training set would be desirable to further improve the accurate segmentation and quantification of stromal stains.

## Conclusions

In summary, the combination of the DLM and MM provides a framework to accurately quantify stromal stains. Starting off with segmenting the stromal compartment as well as the stained region of the stroma, the method developed allows for objective accurate quantification of stromal immunostains. This will help both clinicians and researchers to assess the prognostic implications better and help understand the contribution of the mesenchymal microenvironment to tumour development and progression. The MM uses image registration to register 2 cores stained by different markers (SMA and desmin) in order to detect regions of double staining. In future, such multiplexing with accurate quantification procedures, will help pave research for understanding the functional pathways activated or inactivated together in the tumour-associated stromal compartment.

## Declaration of Competing Interest

The authors declare that they have no known competing financial interests or personal relationships that could have appeared to influence the work reported in this paper.

## References

[1] Kaustav B., Schalper Kurt A., Rimm David L., Vamsidhar V., Anant M. (2019). Artificial intelligence in digital pathology—new tools for diagnosis and precision oncology. Nat Rev Clin Oncol..

[2] Geert L., Sánchez Clara I., Nadya T. (2016). Deep learning as a tool for increased accuracy and efficiency of histopathological diagnosis. Scient. Rep..

[3] Abdelsamea Mohammed M., Alain P., Ruta Barbora G., Justinas B., Arvydas L., Mohammad I. (2019). A cascade-learning approach for automated segmentation of tumour epithelium in colorectal cancer. Exp Syst Appl*.*.

[4] Adel H., Filiz B., Kannappan P. (2008). International Conference on Advanced Concepts for Intelligent Vision Systems.

[5] Alex K., Ilya S., Hinton Geoffrey E. (2012). Imagenet classification with deep convolutional neural networks. Adv Neural Inform Process Syst..

[6] Kaiming H., Xiangyu Z., Shaoqing R., Jian S. (2016). Proceedings of the IEEE Conference on Computer Vision and Pattern Recognition.

[7] Jiayun L., Sarma Karthik V., Chung H.K., Arkadiusz G., Knudsen Beatrice S., Arnold Corey W. (2017). AMIA Annual Symposium Proceedings.

[8] Jun X., Xiaofei L., Guanhao W., Hannah G., Anant M. (2016). A deep convolutional neural network for segmenting and classifying epithelial and stromal regions in histopathological images. Neurocomputing..

[9] Shachi M., Catalin S., Andre K.-B., Rohit B. (2019). Digital assessment of stained breast tissue images for comprehensive tumor and microenvironment analysis. Front Bioeng Biotechnol..

[10] Patrick M., Carina S., Johanna M. (2021). The prognostic impact of the tumour stroma fraction: a machine learning-based analysis in 16 human solid tumour types. EBioMedicine..

[11] Rie N., Satoko M., Hiroko S. (2018). Expression of cancer-associated fibroblast markers in advanced colorectal cancer. Oncol Lett..

[12] Georgia A., Tim C., Price Timothy J. (2011). Desmin expression in colorectal cancer stroma correlates with advanced stage disease and marks angiogenic microvessels. Clin Proteom..

[13] Millar Ewan K.A., Browne Lois H., Julia B. (2020). Tumour stroma ratio assessment using digital image analysis predicts survival in triple negative and luminal breast cancer. Cancers..

[14] Weishaupt Luca L., Jose T., Sophie C.-B. (2021). Deep learning-based tumor segmentation on digital images of histopathology slides for microdosimetry applications. arXiv preprint.

[15] Yue X., Pengfei X. (2021). Global colorectal cancer burden in 2020 and projections to 2040. Translat Oncol..

[16] John C., Gareth T. (2011). The role of tumour stroma in colorectal cancer invasion and metastasis. Cancers..

[17] Hawinkels L.J.A.C., Paauwe M., Verspaget H.W. (2014). Interaction with colon cancer cells hyperactivates TGF-β signaling in cancer-associated fibroblasts. Oncogene..

[18] Mathias U., Cheng Z., Sunjae L. (2017). A pathology atlas of the human cancer transcriptome. Science..

[19] Human Protein Atlas. http://proteinatlas.org.

[20] Olaf R., Philipp F., Thomas B. (2015). International Conference on Medical Image Computing and Computer-Assisted Intervention.

[21] Xiaohao C., Raymond C., Mila N., Tieyong Z. (2017). A three-stage approach for segmenting degraded color images: smoothing, lifting and thresholding (SLaT). J Scient Comput..

[22] George P. (2001). Perceptually uniform color spaces for color texture analysis: an empirical evaluation. IEEE Trans Image Process..

[23] Pluim Josien P.W., Antoine M.J.B., Viergever Max A. (2003). Mutual-information-based registration of medical images: a survey. IEEE Trans Med Imaging..

[24] Nikolas K.J., Johannes K., Pornpimol C. (2019). Predicting survival from colorectal cancer histology slides using deep learning: a retrospective multicenter study. PLoS Med*.*.

[25] Geessink Oscar G.F., Alexi B., Klaase Joost M. (2019). Computer aided quantification of intratumoral stroma yields an independent prognosticator in rectal cancer. Cell Oncol..

[26] Wouter B., Péter B., Jeffrey H. (2019). Epithelium segmentation using deep learning in H&E-stained prostate specimens with immunohistochemistry as reference standard. Scient Rep..

[27] Yves-Rémi V.E., Cédric B., Laurine V., Olivier D., Isabelle S., Christine D. (2018). Segmentation of glandular epithelium in colorectal tumours to automatically compartmentalise IHC biomarker quantification: a deep learning approach. Med Image Anal..

[28] Yves-Rémi VE, Olivier D, Laurine V, Pieter D, Isabelle S, Christine D. High-throughput analysis of tissue-based biomarkers in digital pathology. In 2015 37th Annual International Conference of the IEEE Engineering in Medicine and Biology Society (EMBC):7732–7735. IEEE. 10.1109/EMBC.2015.7320184 26738084

